# Supplementary material for: High HER2 Intratumoral Heterogeneity Is Resistant to Anti-HER2 Neoadjuvant Chemotherapy in Early Stage and Locally Advanced HER2-Positive Breast Cancer
Source: Cancers (Basel). 2025 Jun 24;17(13):2126. doi: 10.3390/cancers17132126 (PMC12248440; doi:10.3390/cancers17132126)
Supplement: Supplementary file 1 [file cancers-17-02126-s001.zip › cancers-3643963-supplementary.pdf]

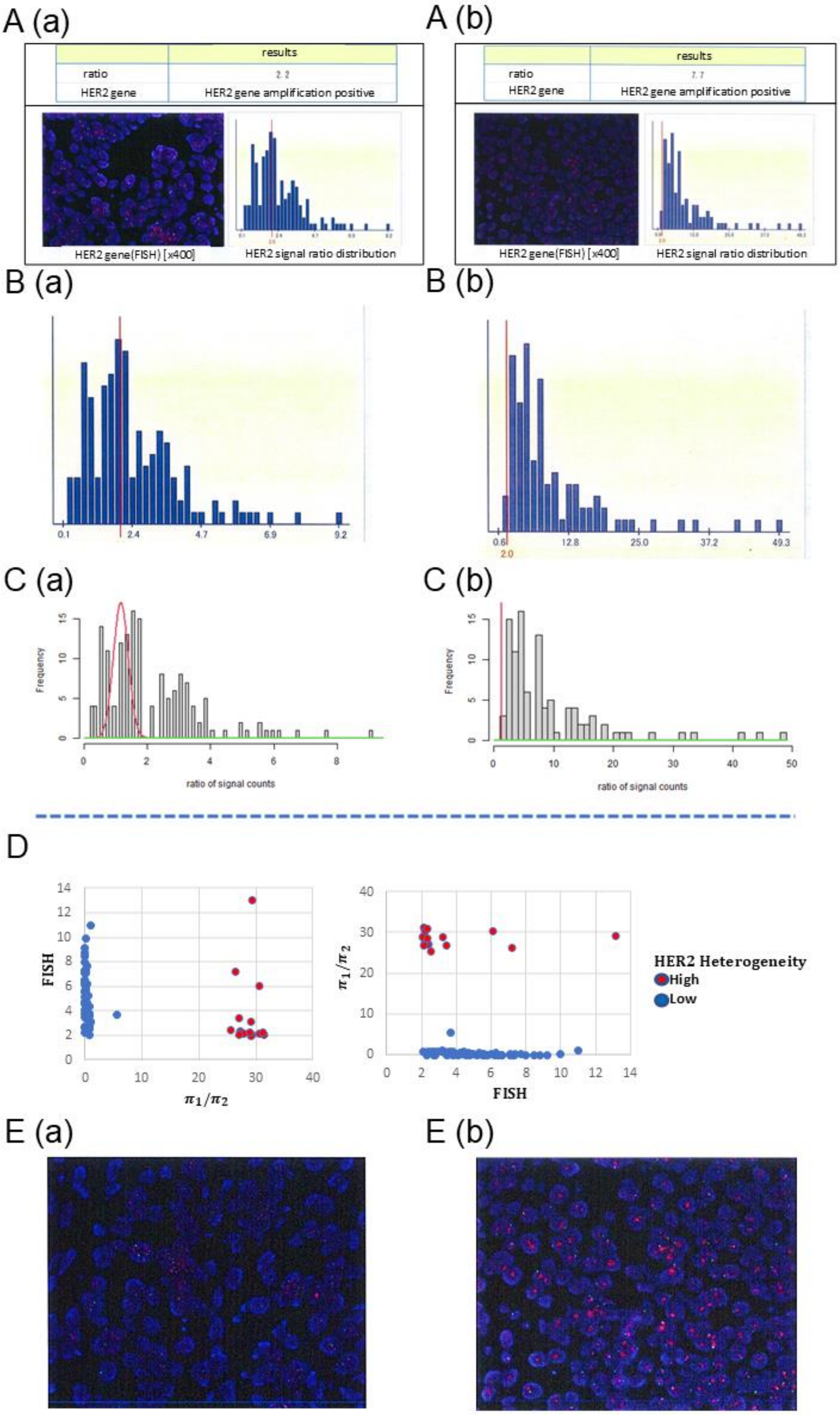

Figure S1: Procedure for diagnostic report visualization. Imaging of HER2 FISH signals in the diagnostic reports (**A**), histogram analysis of HER2 FISH signals (**B**), and fitting with a Gaussian mixture model (**C**), classifying the cases into the HH (a) and LH groups (b). HER2 FISH signals and the mixture ratio (π1/π2) in the overall cohort of patients with HER2-positive breast cancer (**D**). Enlarged figure of HER2/CEP17 signal in Figure S1A (**E**).

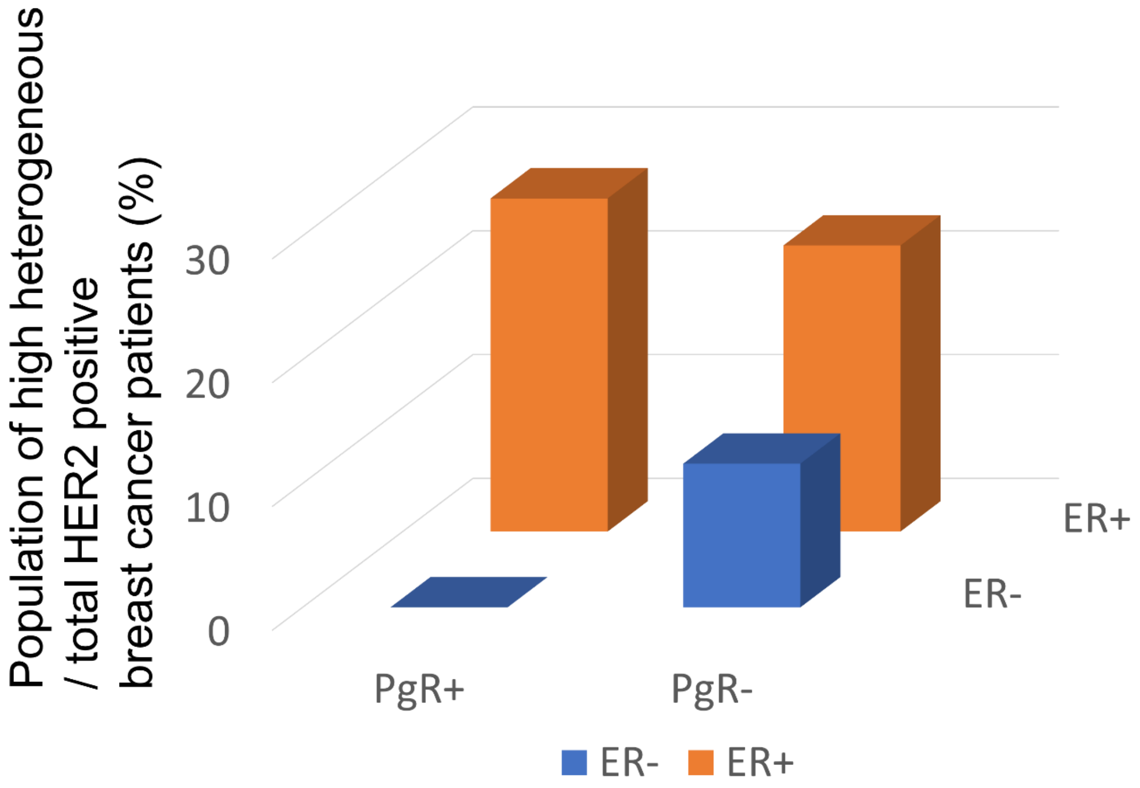

Figure S2: Correlation between the proportion of patients with HER2-positive breast cancer with high heterogeneity (%) and ER/PgR status (n = 97). Tumors with ER and PgR positivity demonstrate the highest HER2 heterogeneity prevalence (ER+ and PgR+, 26.9% [7/26]; ER+ and PgR−, 23.1% [6/26]; ER− and PgR−, 11.6% [5/43]; and ER− and PgR+, 0% [0/2]).
